# Procrastination as a transdiagnostic construct in psychiatric disorders: a conceptual scope review

**DOI:** 10.1192/j.eurpsy.2025.816

**Published:** 2025-08-26

**Authors:** M. S. M. Barbosa, E. L. Guidugli, G. S. M. Barbosa, M. E. G. Melati, N. B. Pagliari, P. B. E. da Silva, P. H. P. Dutra, V. R. Xavier, E. H. Grevet, Y. A. Ferrão

**Affiliations:** 1 Psychiatry, USP-Riberirão Preto, Ribeirão Preto; 2 Psychiatry, UFCSPA, Porto Alegre; 3 Medicine, UNISINOS, São Leopoldo; 4 Medicine, UFCSPA; 5 Psychiatry, UFRGS; 6 Psychiatry, HMIPV, Porto Alegre, Brazil

## Abstract

**Introduction:**

Procrastionation is commonly conceptualized as an irrational, conscious, and sometimes intentional tendency to delay the start or to complete tasks or decisions, despite consequences. The behavior may be present in several psychiatric disorders, especially OCD, ADHD and anxiety disorders However, there is no consensus definition of procrastination, nor established pathophysiology or motivation.

**Objectives:**

To analyze the descriptive psychopathology as well as the presence or absence of associated symptoms, motivations, and consequences of procrastination. In sequence, to conceptualize the construct of “procrastination” based on the literature published and define the motivations and consequences of the behavior for the individuals.

**Methods:**

The scoping review complies with the recommendations of the Preferred Reporting Items for Systematic Reviews and Meta-Analyses (PRISMA) and included articles published English, Spanish and Portuguese, between 1973-2023 and indexed in PubMed/MEDLINE, LILACS, Scielo, EMBASE, Scopus, Web of Science, and PsycINFO database. Research conducted in animals, poster publications, conferences in scientific conclaves, and letters to the author were excluded.

**Results:**

We found 387 studies with different designs (295 [76,2%] were cross-sectional, 12 [3,1%] systematic reviews and 5 [1,3%] were meta-analysis) and published in English (86,6%), Spanish (9,3%) and Portuguese (3,9%). Only 5 studies (1.3%) used populations with AHDH and 1 study (0.02%) used a population with OCD, 7 studies (1.8%) used populations with other disorders (Depression, Anxiety) and 374 studies (96.4%) involved students (mainly university students), workers, or the general population. Of the population the majority was female (63,98%), single, young and has some occupation. In 323 articles, at least one scale was used to assess procrastination, with a total of 40 different ones. Furthermore, 337 studies define procrastination and 305 use the definition of other authors.

**Conclusions:**

Procrastination is a complex event, so far without a defined concept. More investigation is necessary for a more comprehensive understanding of this phenomenon.

**Disclosure of Interest:**

None Declared

